# Potential solution-induced HfAlO dielectrics and their applications in low-voltage-operating transistors and high-gain inverters†

**DOI:** 10.1039/c8ra07813k

**Published:** 2018-10-30

**Authors:** Gang He, Wendong Li, Zhaoqi Sun, Miao Zhang, Xiaoshuang Chen

**Affiliations:** School of Physics and Materials Science, Radiation Detection Materials & Devices Lab, Anhui University Hefei 230601 P. R. China hegang@ahu.edu.cn; Institute of Physical Science and Information Technology, Anhui University Hefei 230601 P. R. China; National Laboratory for Infrared Physics, Chinese Academy of Sciences, Shanghai Institute of Technical Physics 500 Yutian Road Shanghai 200083 P. R. China

## Abstract

Recently, much attention has been paid to the investigation of solution-driven oxides for application in thin film transistors (TFTs). In current study, a fully solution-based method, using 2-methoxyethanol as solvent, has been adopted to prepare InZnO thin films and HfAlO_*x*_ gate dielectrics. Amorphous HfAlO_*x*_ thin films annealed at 600 °C have shown a high transparency (>85%), low leakage current density (6.9 × 10^−9^ A cm^−2^ at 2 MV cm^−1^), and smooth surface. To verify the potential applications of HfAlO_*x*_ gate dielectrics in oxide-based TFTs, fully solution-induced InZnO/HfAlO_*x*_ TFTs have been integrated. Excellent electrical performance for InZnO/HfAlO_*x*_ TFTs annealed at 450 °C has been observed, including a low operating voltage of 3 V, a saturated mobility of 5.17 cm^2^ V^−1^ s^−1^, a high *I*_on_/*I*_off_ of ∼10^6^, a small subthreshold swing of 87 mV per decade, and a threshold voltage shift of 0.52 V under positive bias stress (PBS) for 7200 s, respectively. In addition, time dependent threshold voltage shift under PBS could be described by a stretched-exponential model, which can be due to charge trapping in the semiconductor/dielectric interface. Finally, to explore the possible application in logic operation, a resistor-loaded inverter based on InZnO/HfAlO_*x*_ TFTs has been built and excellent swing characteristic and well dynamic behavior have been obtained. Therefore, it can be concluded that fully solution-driven InZnO/HfAlO_*x*_ TFTs have demonstrated potential application in nontoxic, eco-friendly and low-power consumption oxide-based flexible electronics.

## Abstract 2

Recently, much attention has been paid to the investigation of solution-driven oxides for application in thin film transistors (TFTs).

## Graphical abstract

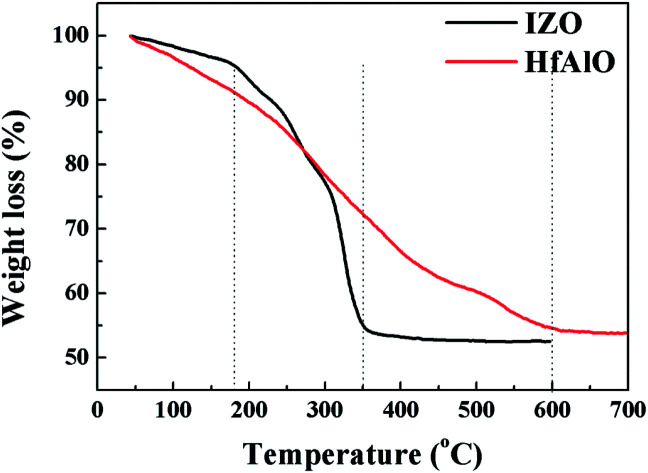

## 1 Introduction

Large-area backplane electronics for upcoming displays have attracted more attention and demonstrated potential applications in next-generation active matrix displays and flexible electronics, such as smart windows, transparent tablets, interactive white boards, and so on.^1,2^ To operate these frontier displays, transparent thin-film transistors (TFTs) with high performance should be built as switching or driving components. Currently, amorphous metal-oxide TFTs have obtained increasing interest owing to the high electron mobility, high transparency, excellent electrical performance for high-speed driving, and solution processability for advanced processing, which is superior to those of the conventional amorphous or poly silicon TFTs.^3–5^ Among the various oxide semiconductor materials, indium–zinc oxide (IZO)-based oxide semiconductor has been considered a promising candidate for the active layer of oxide-based TFTs, exhibiting an optical transmittance of more than 85%, an optical band gap of around 3.5 eV, and high mobility.^6–9^ However, the high power consumption attributed to the high operating voltage impedes the application of oxide-based TFTs.^10–12^ To realize the potential application of oxide-based TFTs in mobile and portable devices, high-*k* gate dielectrics have been selected to enhance the capacitive coupling and reduce the power consumption.

Much effort has been dedicated to the investigation of binary high-*k* dielectric materials, such as ZrO_2_, TiO_2_, and HfO_2_ in the oxide-based TFTs due to their high dielectric constant and wide band gap.^13–15^ Considering their desirable electrical properties and good process compatibility with oxide semiconductors, Hf-based oxide dielectrics have been regarded as good candidates in the display industry.^5,16^ However, the low crystallization temperature and the formation of the grain boundaries for Hf-based high-*k* gate dielectrics contribute to the electrical leakage paths and the increased gate leakage current.^17,18^ The grain boundary of the crystalline structures in the gate insulator, which degrades the electrical performance, is a critical issue. To resolve this issue, amorphous high-*k* dielectrics have been developed as the potential candidates for the optimal operation of the gate insulator in TFTs.

To increase the crystallization temperature, additional dopants, for instance, Si, Ti, and La are introduced into HfO_2_ to form amorphous Hf-based high-*k* oxides.^19–21^ Consequently, the crystalline phase of the Hf-based gate dielectrics were suppressed up to 900 °C.^22^ However, the oxygen diffusion and the reduced dielectric constant after doping degrade the device's performance and prevent its application in TFTs devices. Fortunately, Al incorporating into HfO_2_ worked effectively with increased crystallization temperature and suppressed oxygen diffusion.^23,24^ Furthermore, Al-doped Hf-based oxide showed a considerably low leakage current due to its larger band gap and suitable conduction band offset.^25^ However, the use of vacuum-based deposition method to obtain Al-doped Hf-based gate oxides, such as atomic layer deposition^26^ (ALD) and chemical vapour deposition^27^ (CVD), are limited and incompatible with large-scale production and low cost fabrication. Fortunately, solution-based methods such as inkjet printing, dip-coating, and spin-coating are appropriate for resolving this issue. Operational solution-processed oxide TFTs represent a vital part of this vision, and the stringent requirements on film quality and electrical characteristics make the realization of such TFTs a challenging goal and a key development milestone.^28–30^

In current work, we introduce a simple fabrication process with a large area and low-cost processability for a solution-processed amorphous hafnium–aluminum oxide (HfAlO_*x*_) gate insulator. The correlation between microstructure and dielectric properties of promising amorphous high-*k* HfAlO_*x*_ films has been investigated. The surface morphology and leakage current behaviors of solution processed HfAlO_*x*_ thin films as a function of annealing temperature were examined in terms of its application as gate insulators. To verify the possibility of the solution-derived amorphous HfAlO_*x*_ thin film as gate dielectric in CMOS logics, all-solution-processed IZO-based TFTs and resistor-loaded inverters were also fabricated and examined systematically. Therefore, it can be concluded that the optimized IZO/HfAlO_*x*_ TFTs exhibited optimized electrical performance under a low operating voltage of 3 V, including a high *I*_on_/*I*_off_ of around 6.01 × 10^7^, and a high *μ*_sat_ of 5.17 cm^2^ V^−1^ S^−1^. Meanwhile, the resistor-loaded inverter based on IZO/HfAlO_*x*_ TFT demonstrates full swing characteristics with a gain of 4.32 at 4.0 V.

## 2 Experimental

### 2.1 Precursor solution preparation and characterization

The InZnO precursor solution was prepared by dissolving In(NO_3_)_3_·*x*H_2_O and Zn(NO_3_)_3_·6H_2_O in 2-methoxyethanol. The concentration of IZO precursor solution is 0.1 M and the ratio of In : Zn is 1 : 1. The HfAlO precursor solution was prepared by dissolving HfCl_4_ and Al(NO_3_)_3_·9H_2_O in 2-methoxyethanol. The purity of all the starting materials is 99.9% and purchased from Aladdin pharmaceuticals. The concentration of HfAlO precursor solution is 0.1 M and the ratio of Hf : Al is 2 : 1. All precursor solutions were stirred vigorously in 600 rpm for 6 h at room temperature. The thermal behaviors of InZnO and HfAlO xerogel were measured using a thermal-gravimetric analyzer (TGA) with a heating rate of 10 °C min^−1^ (STA449F3) in air.

### 2.2 Thin film fabrication and characterization

The substrate was heavily doped p-Si (0.0015 Ω cm) and acted as gate electrode. The substrates were ultrasonic cleaned in acetone, alcohol and deionized water in sequence for 10 min. The substrates were treated by oxygen plasma for 10 min to enhance hydrophilicity before deposition. The HfAlO solution was filtered through a 0.22 μm syringe filter and spun on the substrates at 5000 rpm for 20 s. Then the films were baked at 150 °C for 10 min and repeat previous procedure once. After deposition, the samples were treated by ultraviolet (UV) light for 30 min and annealed at 300, 400, 500 and 600 °C for 1 h in air. The power of UV lamp was 1 kW and the UV lamp was 20 cm from the samples. The microstructures of HfAlO_*x*_ thin films were investigated by X-ray diffractometer (XRD, MXP 18AHFMAC Science, Yokohama). The absorbance and transmittance of HfAlO_*x*_ films deposited on quartz substrate were measured by a UV-Vis spectrophotometer (Shimadzu, UV-2550). The surface morphologies of HfAlO were measured by using atomic force microscope (AFM, Smart Lab, Multimode 8). The chemical compositions of HfAlO_*x*_ were analyzed by X-ray photoelectron spectroscopy (XPS, ESCALAB 250Xi). C 1s peak at 284.6 eV was taken as a reference for charge correction. The charge neutralizations of X-ray bombarded samples were performed by flood guns and spectral deconvolution was performed by Shirley background subtraction using a Voigt function convoluting Gaussian and Lorentzian functions. The thicknesses of HfAlO_*x*_ and IZO thin films were measured by spectroscopic ellipsometry (SE, SANCO Co, Shanghai, SC630). A MOS structure of Al/HfAlO/p^+^-Si was used to measure the dielectric properties of HfAlO films by an impedance analyzer (Agilent 4294A).

### 2.3 TFTs fabrication and characterization

The InZnO solution was spun on the HfAlO_*x*_ films at 5000 rpm for 20 s. Then the samples were annealed at 350, 400, 450 and 500 °C for 1 h in air. Finally, the Al source and drain electrodes were deposited on the films *via* a shadow mask. The channel length and width were 100 and 1000 μm. In addition, the SiO_2_-based TFTs were preparation for comparison. The 200 nm-thick SiO_2_ was obtained by heavily doped p-Si with the thermal oxidation procedure. The detailed schematic diagram of the solution-derived IZO and HfAlO_*x*_ thin films and In_2_O_3_-based TFTs fabrication are demonstrated in Fig. S1 (ESI).† The electrical properties of the integrated TFTs were measured by using semiconductor parameter analyzers (Keithley 2636B; Agilent B1500A) in a dark box. The saturation mobility (*μ*_sat_) was extracted from transfer characteristics using the following equation^31^
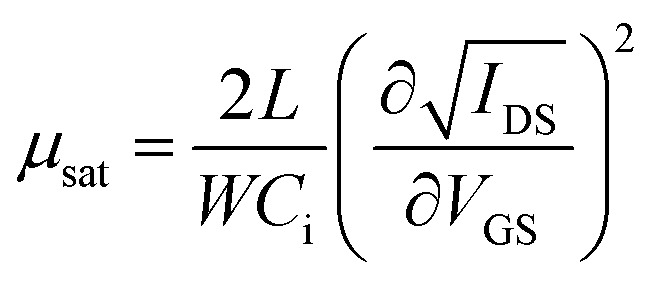
where *C*_i_ is the areal capacitance of the gate dielectric, *W* and *L* are the channel width and length of the TFT, *V*_G_ is the gate voltage and *V*_TH_ is the threshold voltage, which was determined in the saturation region by linear fitting *I*_D_^1/2^*vs. V*_G_ plot. The density of interface states (*D*_it_) can be inferred using the following equation,^31^
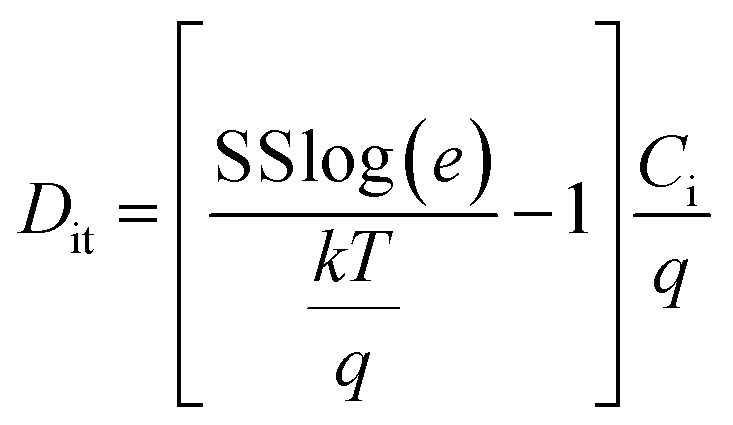
where *k*, *T*, and *q* are Boltzman's constant, absolute temperature, and charge quality, respectively.

## 3 Results and discussion

### 3.1 Microstructure analysis and surface morphology of solution-derived HfAlO_*x*_ thin films

**Fig. 1:**
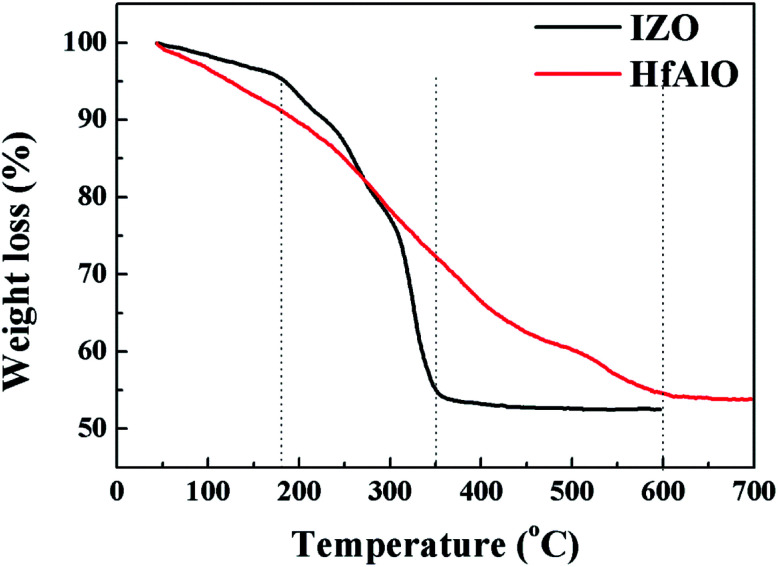
Thermal behavior of the solution-induced IZO and HfAlO_*x*_ xerogel.

To investigate the thermal behavior of IZO and HfAlO_*x*_ thin films, TGA measurement of HfAlO_*x*_ and IZO precursor solutions was performed with a heating rate of 10 °C min^−1^ and the experimental result is demonstrated in Fig. 1. For IZO xerogel, the initial decrease of weight for IZO below 180 °C is attributed to the decomposition of the residual nitrate species originating from the metal nitrate salt.^32^ The continuous decreased weight between 180 °C to 350 °C can is mainly due to the dihydroxylation.^33^ When the temperature exceeds 350 °C, the reduction in mass has not been observed, suggesting that complete transformation from xerogel to IZO oxide has been completed. For HfAlO_*x*_ xerogel, the transformation in the decomposition process of HfAlO_*x*_ xerogel is not similar to IZO xerogel. It could be noted that the dehydroxylation and alloy reaction begin at the initial stage. A more pronounced, gradual mass loss over several hundred degrees have been detected. Conversion of the HfAlO precursor to the corresponding oxide is completed by 600 °C. Based on this dehydroxylation behavior, the annealing temperature range for clear gate switching modulation of successful oxide TFTs is estimated to range from 350 °C to 600 °C.

**Fig. 2:**
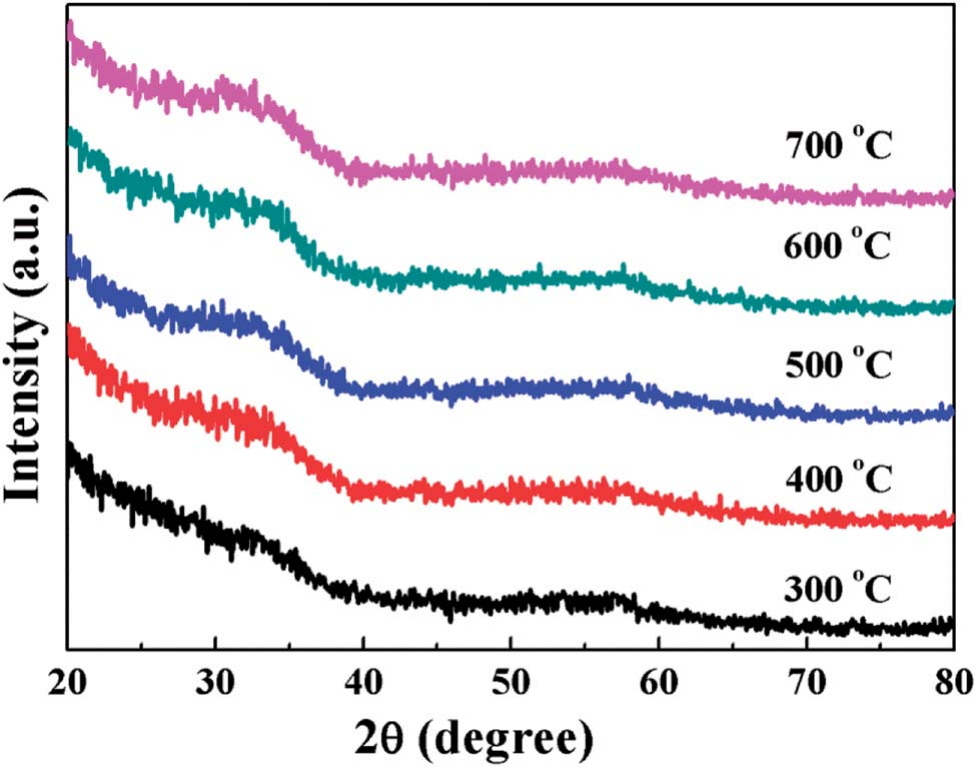
Annealing temperature dependent XRD patterns of HfAlO_*x*_ films.

The evolution of the microstructure of the spin-coated HfAlO_*x*_ films annealed at various temperatures was investigated by XRD measurements and the results are demonstrated in Fig. 2. No apparent diffraction peaks of crystalline phase in XRD patterns have been observed in the films regardless of the annealing temperature, indicating that the HfAlO_*x*_ films are identified as amorphous phase. It is confirmed further that introducing appropriate amount of Al_2_O_3_ in HfO_2_ would remarkably block the crystallization of the HfO_2_ films and lead to the increase of crystallization temperature of HfO_2_, which is in good agreement with our previous investigation.^34^ Being a dielectric layer for the bottom-gated TFTs, the amorphous phase is more suitable than the crystalline phase owing to its smooth surface, low leakage current, and high breakdown electric field. According to the grain-boundary charge-trapping model,^35^ the grain boundary usually acts as the trapping and scattering center in polycrystalline film, leading to the high leakage current and the degraded insulating reliability.^33^ Such dielectrics certainly show high off-state current in TFT devices. In addition, the amorphous thin films generally exhibit smoother surface roughness compared with the crystalline ones, which is a prerequisite for expeditious charge carrier mobility in the TFT devices.^36^

**Fig. 3:**
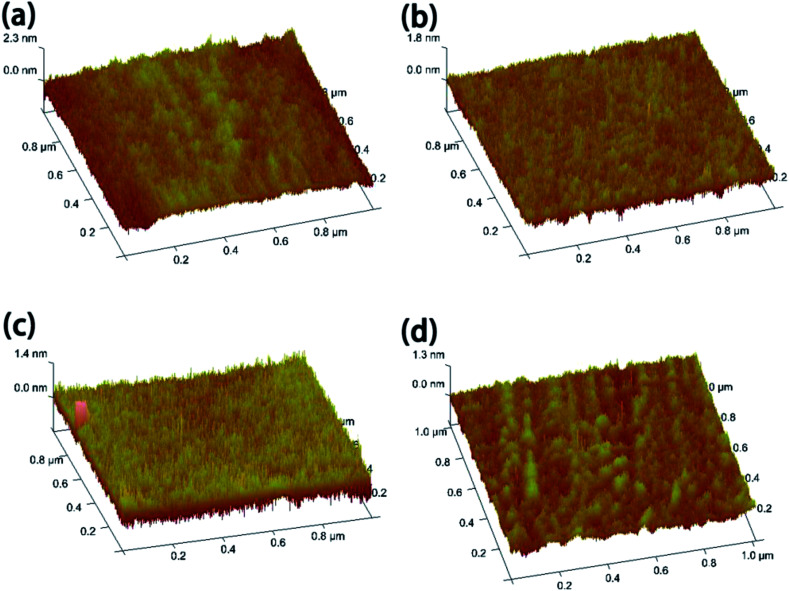
Surface morphologies of (a) 300 °C, (b) 400 °C, (c) 500 °C and (d) 600 °C-annealed HfAlO_*x*_ films.

It is well known that high-performance TFTs should have dielectric layers with smooth surface as the carrier transport is affected by the interface between the channel and the dielectric layers. To investigate the evolution of the surface morphology of HfAlO_*x*_ films as a function of annealing temperature, AFM images of the solution-derived HfAlO_*x*_ films are shown in Fig. 3. The root-mean-square (RMS) values of HfAlO films annealed at 300, 400, 500 and 600 °C are 0.266, 0.189, 0.205, and 0.147 nm, respectively. It is obvious to see that RMS values smaller than 1 nm are observed for solution-processed HfAlO_*x*_ dielectrics, which is conductive to the growth of high-quality channel layer and improve the TFTs' performance. The smooth surface of the HfAlO films is not only attributed to their amorphous structure, but also to the use of UV annealing treatment. Based on the investigation from Tak *et al.*, it can be noted that UV treatment consists of three main reactions (Fig. S2†). First, physical bonds are effectively converted into chemical bonds. It is proposed that the energy provided by UV light is sufficient to decompose residual weak chemical bonds in spin-coated oxide films, and the simultaneous thermal treatment induces the reorganization and rearrangement of the decomposed chemical bonds into strong chemical bonds. Second, UV treatment promoted the oxidation of the gate dielectric films and led to a lower number of uncoordinated oxygen species. Third, the increased surface energy of the oxide films indicates highly chemically reactive states and improved surface smooth. Therefore, it can be concluded that UV treatment can effectively accelerate the decomposition of organic ligands, reduce the nanopores in the films, and attribute the increased surface energy and the decreased surface roughness.^17,37,38^

### 3.2 Optical properties analysis of solution-derived HfAlO_*x*_ thin films

**Fig. 4:**
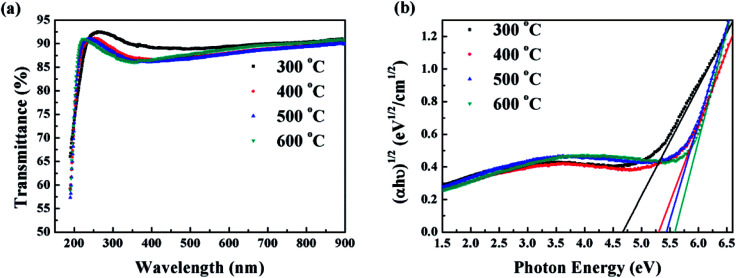
(a) Optical transmittance and (b) band gap of HfAlO_*x*_ films annealed at different temperature.

To investigate the optical properties of the HfAlO_*x*_ films, transmittance and absorbance of the HfAlO_*x*_ films were measured. Fig. 4 shows the optical transmittance and absorbance spectra of HfAlO_*x*_ films as a function of annealing temperature. All the samples demonstrate high average optical transmittance values (>85%) in the visible region, indicating that HfAlO_*x*_ films can be considered as candidate dielectric materials for transparent devices. By using a standard Tauc plot method, the optical bandgap (*E*_g_) is calculated and the results are displayed in Fig. 4b. It can be seen that the *E*_g_ value increases from 4.76 to 5.57 eV when annealing temperature changes from 300 to 600 °C. Apparently, blue shift in band gap has been detected with the increase of the annealing temperature. For the low-temperature-driven HfAlO_*x*_ thin film, the presence of defects in the thin film would produce localized states in the bandgap, leading to the reduced bandgap energy of the film. After high temperature annealing, the annihilation of oxygen vacancies/defects results in a decrease of the density of states in the band structure, which leads to an increased bandgap energy of the HfAlO_*x*_ thin films.^28^ The increased *E*_g_ is beneficial for suppressing the carrier transport from channel to gate dielectric.

### 3.3 Chemical bonding states of HfAlO_*x*_ thin films

**Fig. 5:**
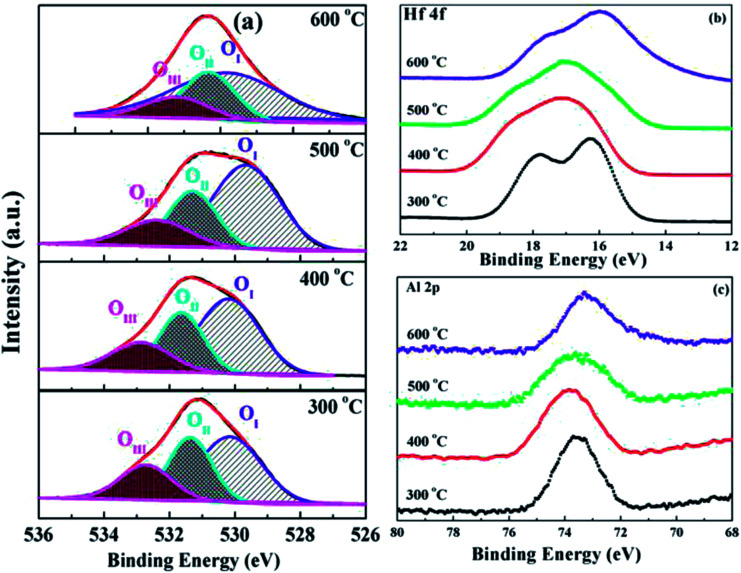
XPS spectra of O 1s (a), Hf 4f (b), and Al 2p (c) peaks for HfAlO_*x*_ dielectrics as a function of annealing temperature.

The chemical bonding states and compositions of sputtering-derived HfAlO_*x*_ thin films were analyzed by XPS. Fig. S3† displays the survey spectra of the HfAlO_*x*_ thin films as a function of annealing temperature. It can be noted that Hf, Al, O, and C have been detected, suggesting that all the films are free from other contaminations and Al is successfully incorporated into the HfO_2_ gate dielectrics. O 1s XPS spectra for HfAlO_*x*_ thin films have been demonstrated in Fig. 5a and deconvoluted O 1s spectra centered at 530.14 eV, 531.36 eV and 532.73 eV, have been observed, respectively. The peak located at 530.14 eV is assigned to the metal–oxygen bond (O_I_) in HfAlO_*x*_ lattice and the peak located at 531.36 eV is attributed to the oxygen vacancy (O_II_) in lattice.^39,40^ The peak centered at 532.73 eV may be related to loosely bound oxygen on the surface of films, such as adsorbed H_2_O and –OH.^41^ Increasing the annealing temperature from 300 to 600 °C, the fraction of O_I_ in HfAlO_*x*_ increases and O_II_ and O_III_ species decrease, which indicates that high temperature annealing reduces the oxygen vacancy and hydroxyl species, and improves the metal–oxygen lattice. The presence O_II_ and O_III_ generally creates trap and defect states in the band gap of the dielectric film, leading to the increased leakage current and the reduced breakdown voltage.^42^ Therefore, higher temperature annealing is effective in controlling the oxygen vacancy and bonded oxygen content and obtain high quality high-*k* gate dielectric for application in TFT devices. To obtain more information on chemical bonding states from HfAlO_*x*_ thin films, the evolution of the Hf 4f and Al 2p core-level XPS spectra related to annealing temperature has been investigated, as shown in Fig. 5b and c. Compared to HfO_2_, the doublet peaks corresponding to Hf 4f_5/2_ and Hf 4f_7/2_ for HfAlO_*x*_ thin films shift towards higher binding energy sides, indicating the formation of Hf-aluminate.^34^ However, for the 600 °C-annealed sample, the shift of binding energy in Hf 4f peak towards lower energy side has been detected, which can be due to the decomposition of HfAlO_*x*_ and partial formation of HfO_2_. The same trend has been observed for Al 2p core-level XPS spectra.

### 3.4 Dielectric and electrical properties of solution-derived HfAlO_*x*_ thin films

**Fig. 6:**
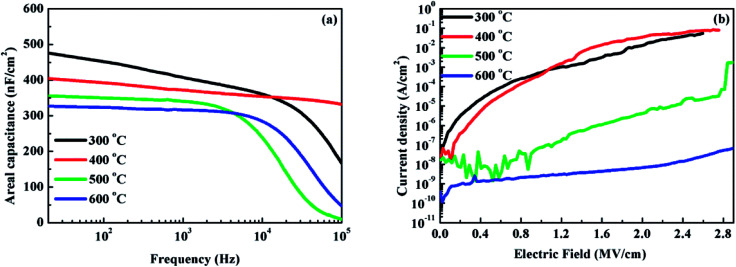
(a) Capacitance–frequency curves and (b) leakage current density–electric field curves of Al/HfAlO/p^+^-Si capacitor.

To investigate the annealing temperature dependence on the dielectric and electrical properties of the solution-driven HfAlO_*x*_ thin films, MOS capacitors based on Al/HfAlO/p^+^-Si were constructed. Fig. 6a shows the areal capacitance (*C*) as a function of frequency (*f*) for HfAlO_*x*_ capacitors. The areal capacitance of HfAlO capacitors annealed at 300, 400, 500 and 600 °C is 475, 404, 355, and 326 nF cm^−2^ at 20 Hz, respectively. It can be noted that reduction in the areal capacitance has been observed with the annealing temperature increasing from 300 to 600 °C. Generally speaking, water molecules can be absorbed by polarizable hydroxyl groups. Previous XPS results have confirmed the existence of the large number of polarizable hydroxyl groups in low-temperature-processed HfAlO_*x*_ thin films. Therefore, absorbed water molecules would lead to the high capacitance owing to the high dielectric constant of water molecules.^43^ At the same time, the slight decreases for the areal capacitance in the high frequency range can be attributed to the limited polarization response time.^44^ To evaluate the leakage behavior of the HfAlO_*x*_ thin films, the corresponding leakage current densities and electric field characteristics of MOS capacitors annealed at different temperatures have been shown in Fig. 6b. For the low-temperature-annealing sample, the relatively large leakage current density may be attributed to the incomplete decomposition of hydroxyl groups. The reduced leakage current density has been observed with the increase in the annealing temperature, which probably originates from the gradually decomposition of residuals and the reduced defect density. For the 600 °C-annealed sample, the lowest leakage current density of 6.0 × 10^−9^ A cm^−2^ at 2 MV cm^−1^ has been achieved. As a result, it can be inferred that the excellent electrical performance for 600 °C-annealed solution-processed HfAlO_*x*_ dielectric thin films guarantees its potential application in low-voltage transistor.

### 3.5 Solution-driven IZO TFTs based on HfAlO_*x*_ dielectric

**Fig. 7:**
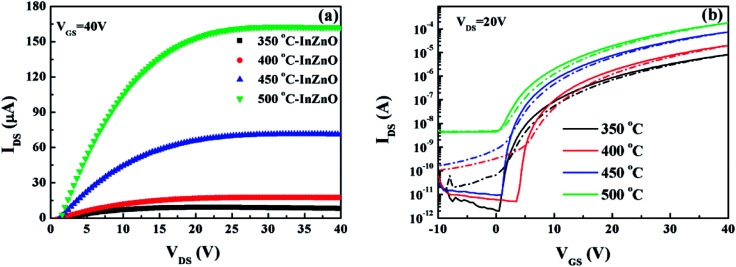
(a) Output and (b) transfer curves of IZO/SiO_2_ TFTs as a function of annealing temperature.

Based on above analysis, it can be noted that the 600 °C-processed HfAlO_*x*_ gate dielectric completely meets the application requirements of TFTs. Before investigating the feasibility of solution-processed HfAlO_*x*_ as gate dielectric in TFTs, the possibility of solution-derived IZO/SiO_2_ TFTs with bottom-gate and top-contact architecture was evaluated. The solution-processed IZO channel layers were annealed at 350–500 °C. The output curves of each IZO/SiO_2_ TFTs are shown in Fig. S4† and the annealing temperature dependent output characteristics of the IZO/SiO_2_ TFTs, at a gate voltage of (*V*_GS_) of 40 V, are depicted in Fig. 7a. The low saturation current at 350 °C may mainly attributed to the existed hydroxyl groups and the formation of defect states. When increasing the annealing temperature, the increased saturation current has been observed, indicating the reduced lattice defects, such as hydroxides and residual impurities.^45^ The representative transfer characteristics of In_2_O_3_ TFTs, at a drain voltage of (*V*_DS_) of 20 V, are displayed in Fig. 7b. The *μ*_sat_ and threshold voltage (*V*_TH_) in the saturation region were determined from linear fits to the dependence of the square root of *I*_D_ on *V*_G_. The subthreshold swing (SS) was extracted from the linear portion of a plot of the log *I*_D_*versus V*_G_. The extracted key TFTs performance parameters as a function of annealing temperature are summarized in Table 1. It can be seen that *μ*_sat_ values increase from 0.15 to 3.08 cm^2^ V^−1^ S^−1^ with the increase in annealing temperature, which can be due to the decomposition of organic groups and the formation of metal–oxygen bonds. Meanwhile, increase in *I*_on_/*I*_off_ and reduction in SS have been detected with the increased annealing temperature, which can be attributed to the reduced oxygen vacancy and free carrier concentration. However, further increasing the annealing temperature for IZO channel layer to 500 °C results in the degraded device performance, originating from the increased trap states near the IZO/SiO_2_ interface, confirmed by the *D*_it_ values shown in Table 1. On the basis of the extracted electrical parameters, it can be noted that the solution-derived 450 °C-annealed IZO/SiO_2_ TFTs demonstrates the optimized electrical performance, including a high *μ*_sat_ of 1.41 cm^2^ V^−1^ S^−1^, a high *I*_on_/*I*_off_ value of 10^6^, and a small SS value of 0.507 V dec^−1^, respectively. However, all the TFTs devices operate at high voltages and hence increase the power consumption due to the low dielectric constant for SiO_2_. To decrease the operation voltage and reduce the power consumption of solution-derived IZO TFTs, high-*k* gate dielectric should be explored.

**Table 1:** Electrical parameters of IZO/SiO_2_ TFTs annealed at different temperature

	*V*_TH_ (V)	Δ*V*_TH_ (V)	*I*_on_/*I*_off_	*μ* (cm^−2^ V^−1^ s^−1^)	SS (mV per decade)	*N*_T_ (cm^−2^)
350 °C	13.5	2.5	4 × 10^6^	0.15	633	9.57 × 10^11^
400 °C	15.0	3.2	4 × 10^6^	0.40	646	9.78 × 10^11^
450 °C	13.9	2.2	8 × 10^6^	1.42	507	7.46 × 10^11^
500 °C	13.0	2.6	4 × 10^4^	3.08	2469	4.02 × 10^12^

In order to validate the usefulness of high-*k* HfAlO_*x*_ film as gate insulators for TFTs, bottom-gated fully solution-derived IZO/HfAlO_*x*_ TFTs were fabricated. Previous 600 °C-annealed HfAlO_*x*_ has been adopted to act as the dielectric layer due to its relatively low leakage current and good dielectric properties. A solution-processable IZO precursor solution was spin-coated on a HfAlO_*x*_/Si stack, followed by annealing from 350 to 500 °C to pursue the optimized TFTs device performance.

**Fig. 8:**
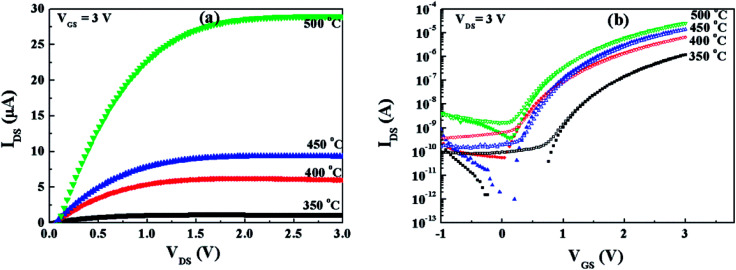
(a) Output and (b) transfer curves of IZO/HfAlO TFTs as a function of annealing temperature.

Fig. S5† displays the output curves of each TFT and the summarized output curves of IZO/HfAlO_*x*_ TFTs at a *V*_GS_ of 3 V are shown in Fig. 8a. It can be seen that the output characteristics of IZO/HfAlO_*x*_ TFTs exhibit typical n-channel conduction behavior with clear pinch-off voltage and current saturation. By comparing the transfer curves of IZO/HfAlO_*x*_ TFTs with IZO/SiO_2_ TFTs, it can be seen that the off-current region increases with decreasing *V*_GS_ for the IZO/HfAlO_*x*_ TFTs while the IZO/SiO_2_ TFTs remains flat, which can be due to the smaller band gap of HfAlO_*x*_ compared with SiO_2_, and thus, the carrier injection into the HfAlO_*x*_ will be much easier than into SiO_2_. Interestingly, by replacing SiO_2_ with high-*k* HfAlO_*x*_ thin film, the operation voltage drastically reduces from 40 V to 3 V. As a result, the HfAlO_*x*_/IZO TFTs expend lower consumption than the SiO_2_/IZO TFTs. Because the field-induced current is proportional to the field-induced charge density, a reasonable technique to achieve low-voltage operation in TFT is to use HfAlO_*x*_ as the gate dielectric, which can afford greater surface charge density at the semiconductor/dielectric interface. Fig. 8b shows the representative transfer characteristics of TFTs based on 600 °C-annealed HfAlO_*x*_ films as a function of the annealing temperature. The extracted electrical characteristics, including the *μ*_sat_, *I*_on_/*I*_off_, *V*_TH_, *D*_it_, and SS, of HfAlO_*x*_/IZO TFTs with different annealing temperatures were summarized in Table 2. With the increase of the annealing temperature from 350 to 450 °C, *V*_TH_ and SS decrease from 1.63 and 120 to 1.14 V and 87 mV dec^−1^, while *I*_on_/*I*_off_ and *μ*_sat_ increase from 10^4^ and 0.80 to 10^6^ and 9.50 cm^2^ V^−1^ S^−1^, respectively. Apparently, the optimized electrical performance has been achieved for 450 °C-annealed HfAlO_*x*_/IZO TFTs.

**Table 2:** Electrical parameters of IZO/HfAlO TFTs annealed at different temperature

	*V*_TH_ (V)	Δ*V*_TH_ (V)	*I*_on_/*I*_off_	*μ* (cm^−2^ V^−1^ s^−1^)	SS (mV per decade)	*N*_T_ (cm^−2^)
350 °C	1.63	0.06	∼10^4^	0.80	120	1.98 × 10^12^
400 °C	1.23	0.10	∼10^4^	2.52	177	3.86 × 10^12^
450 °C	1.14	0.19	∼10^6^	5.17	87	8.93 × 10^11^
500 °C	1.16	0.09	∼10^5^	9.50	183	4.06 × 10^12^

In the solution-derived channel layer thin films, oxygen vacancies form when dehydroxylation and polycondensation occur. Thus, it is expected that annealing at a higher temperature leads to the reduction of oxygen vacancy and the formation of more metal–oxygen bonds. As a result, *V*_TH_ shifts towards negative direction as the annealing temperature increases due to the reduced interfacial defects acting as the carrier trap at IZO/HfAlO_*x*_ interface. With increasing the annealing temperature, the oxygen vacancy and free carrier concentration decrease. As a result, *I*_on_/*I*_off_ initially increases because of the reduced *I*_off_. As we know, a small SS is expected to achieve a high operation speed and low power consumption. Normally, the SS value is dependent on the traps located in channel/dielectric interface.^37^ With the increase in annealing temperature, the reduction in SS value may be attributed to the large areal capacitance of the HfAlO_*x*_ dielectric layer and the electronic-clean interface between IZO and HfAlO_*x*_. Based on Jeong's report,^46^ it can be observed that the conduction band minimum in the metal oxide semiconductors is primarily composed of dispersed vacant s states with short interaction distances for efficient carrier transportation, which can be achieved in ionic oxide but not obviously in hydroxide. Therefore, higher temperature annealing accelerates the decomposition of –OH groups and the alloy reaction and leads to the formation of metal–oxygen framework, which contributes fewer defects in both bulk and interface. For the top-gated TFTs, the carrier transport is limited in a narrow region at channel/dielectric interface. In this regard, the reduced defects at the IZO/HfAlO_*x*_ interface could achieve the rapid transport of the induced carriers, and thus enhanced *μ*_FE_ would be expected.

From Table 2, it can be noted that the *D*_it_ values of IZO/HfAlO_*x*_ TFTs annealed at 350, 400, and 450 °C are calculated to be 1.98 × 10^12^, 3.86 × 10^12^, and 8.93 × 10^11^ cm^−2^, respectively. A large *D*_it_ has been observed in the 350 and 400 °C-annealed TFTs, which is attributed to the incomplete decomposition of residual organic groups and the existence of the defects states near IZO/HfAlO_*x*_ interface. Smallest *D*_it_ has been obtained for 450 °C-annealed sample, which is lower than that of TFTs based on spin-coated MgO (1.1 × 10^13^ cm^−2^)^47^ and water-induced ScO_*x*_ (1.1 × 10^13^ cm^−2^).^48^ Such a small *D*_it_ is not only beneficial to carrier transport in the interface region, but also to the operation stability.

In spite of the high saturation current and the large *μ*_sat_ for 500 °C-annealed IZO/HfAlO_*x*_ TFTs, the degradation in some important electrical performance has been observed. *D*_it_ for 500 °C-annealed IZO/HfAlO_*x*_ TFTs is calculated to be 4.06 × 10^12^ cm^−2^, approaching nearly one magnitude larger than that of IZO/HfAlO_*x*_ TFTs, which can be attributed to the increased trap states near the IZO/HfAlO_*x*_ interface. Meanwhile, the reduced *I*_on_/*I*_off_ value for 500 °C-annealed TFTs is mainly caused by the large off-state current (*I*_off_), which will lead to the inevitable static power consumption and degrade device performance.^49^ It is known that static power consumption is comparable to dynamic power in modern silicon chips or even become dominating in the future.^50^ Therefore, the *I*_off_ has been regarded as a critical parameter to evaluate the power consumption of a device in modern integrated circuits.

### 3.6 Bias stability characterization for solution-driven IZO TFTs

**Fig. 9:**
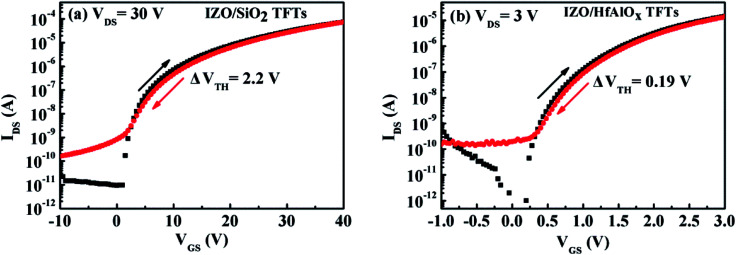
Transfer characteristics of (a) IZO/SiO_2_ TFTs and (b) IZO/HfAlO_*x*_ TFTs.

The operational device stability of a given FET is often characterized by the amount of hysteresis between the forward and reverse sweep. Based on Fig. 9, it can be noted that clockwise hysteresis phenomena (Δ*V*_TH_ = 2.2 V for IZO/SiO_2_ FETs and Δ*V*_TH_ = 0.19 V for IZO/HfAlO_*x*_ TFTs) have been observed for both TFTs, indicating that accumulated electrons are trapped in the defect states located at dielectric/channel interface.^51^ During the forward sweeping of the gate voltage, some of the accumulated electrons are transferred into the unoccupied surface states. When the gate voltage is swept back, these states remain filled until the trapped electrons are thermally detrapped, which leads to the clockwise hysteresis. Compared to IZO/SiO_2_ FETs, the hysteresis in IZO/HfAlO_*x*_ TFTs (see Fig. 9b) is reduced substantially, indicating the reduction in the interfacial trap states by the introduction of a high-*k* HfAlO_*x*_ dielectric, which is consistent with the previous *D*_it_ value.

**Fig. 10:**
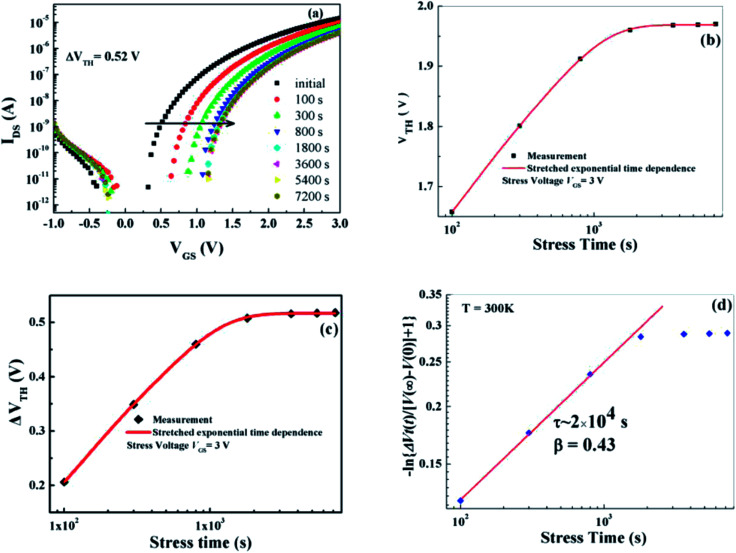
(a) Transfer curves of 450 °C annealed IZO/HfAlO_*x*_ TFTs under PBS test. (b) The *V*_TH_ as a function of stress time. (c) The time dependence of Δ*V*_TH_ in the IZO/HfAlO TFTs under the bias stress of 3 V. (d) Threshold voltage shifts during bias stress at *T* = 300 K on a log_10_[−ln{Δ*V*_*t*_(*t*)/[*V*(∞) − *V*(0)] + 1}] *vs.* log_10_(*t*) plot. The data have been fitted to a stretched exponential {1 − exp[−(*t*/*t*_o_)^*β*^], which reads a straight line in this format.

To achieve manufacturability of IZO-based TFT, it is crucial to solve the problems of the threshold-voltage (*V*_TH_) shift of the transistor with time under prolonged bias. Any shift in the threshold voltage of the driving transistor under gate and drain-bias stress conditions will cause a change in its output drain current, leading to circuit malfunction. Thus, device degradation due to bias-induced instability is a critical issue that must be solved. To investigate the bias stability of the IZO/HfAlO_*x*_ TFTs, positive bias stress (PBS) tests were performed with the source and drain connected to the ground (*V*_DS_ = 0 V). Fig. 10a shows the evolution of typical transfer characterization of the 450 °C-annealed IZO/HfAlO_*x*_ TFTs subjected to a positive gate-bias voltage of 1 V for different stress time at room temperature (*T* = 25 °C). It can be observed that a parallel shift of the transfer curve toward the positive direction, and the evolution of the shift is from rapid to slow with stress time increasing, indicating there is no additional defect creation at the channel/dielectric interface during bias stressing.^4^ After the transistor has undergone the gate-bias stressing, the negligible change in the SS and carrier mobility suggests that the creation of extra electron trapping states at the semiconductor/dielectric interface is not significant. At the same time, higher operation stability with a small threshold voltage shift (Δ*V*_TH_) of 0.52 V up to 7200 s for 450 °C-annealed IZO/HfAlO_*x*_ TFTs has been detected, revealing that there are a small number of defects at the IZO/HfAlO_*x*_ interface, which is confirmed by previous *D*_it_ data. The *V*_TH_ is presented as a function of stress time on a logarithmic scale in Fig. 10b. Based on Fig. 10b, it can be noted that *V*_TH_ is shifted significantly at the beginning of the bias stressing, and as the stress continues, *V*_TH_ approaches a saturation value. The shift of *V*_TH_ is not accompanied by the SS degradation (Fig. 10a), which indicates that the Δ*V*_TH_ in IZO is attributed to the trapping of electrons in the interface or bulk dielectric layers with negligible creation of additional interface traps.^52^

The investigation of the time dependence of Δ*V*_TH_ can be used to confirm the dominant charge trapping mechanism causing the bias stress-induced Δ*V*_TH_ in TFTs.^53^ To further investigate the bias stress-induced threshold voltage shift phenomenon in IZO/HfAlO_*x*_ TFTs, the stress time dependences of Δ*V*_TH_ under positive gate-bias voltage of 1 V at room temperature is examined and shown in Fig. 10c. The Δ*V*_TH_ is defined as Δ*V*_TH_ = *V*_TH,*t*_ − *V*_TH,i_, where *V*_TH,*t*_ is the *V*_TH_ value at the measured time and *V*_TH,i_ is the initial *V*_TH_. From Fig. 10c, it can be seen that the threshold voltage shifts rapidly and then is saturated quickly with the increase of stress time. Previous investigation on the bias stress-induced degradation of IGZO-based TFTs have indicated that the time dependence of Δ*V*_TH_ under bias stresses in IGZO TFTs is followed by a logarithmic time-dependence model,^53^ but current work shows that the time dependence of Δ*V*_TH_ can be fitted well with a stretched-exponential equation for all stress conditions, which has been developed to model the Δ*V*_TH_ based on the charge trapping mechanism in α-Si TFT with high-*k* dielectric.^52^ The stretched exponential model of Δ*V*_TH_ is defined as^53^
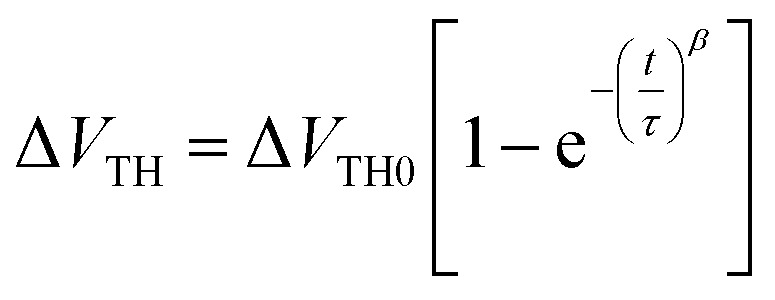
where Δ*V*_TH0_ is the Δ*V*_TH_ at infinite time, approximating the voltage drop across the insulator; *t* is the stress time; *τ* is the characteristic detrapping time of carriers, and *β* is the stretched exponential exponent. In Fig. 10d, typical bias–stress curves for *V*_GS_ = 3 V, *T* = 300 K are plotted as −ln{Δ*V*_*t*_(*t*)/[*V*(∞) − *V*(0)] + 1} over *t* on a double-logarithmic scale, where *V*(∞) is the applied gate bias and *V*(0) is the initial threshold voltage. In this format, a stretched exponential dependence gives a straight line. From the linear fits, a characteristic trapping time (*τ*) of 2 × 10^4^ s and a stretched exponential constant (*β*) of 0.43 have been determined. These results are consistent with those for InGaZnO-based TFT, where charge trapping phenomenon is also considered as the dominant mechanism of bias-stress-induced Δ*V*_TH_.^54^

### 3.7 Resistor-loaded inverter

**Fig. 11:**
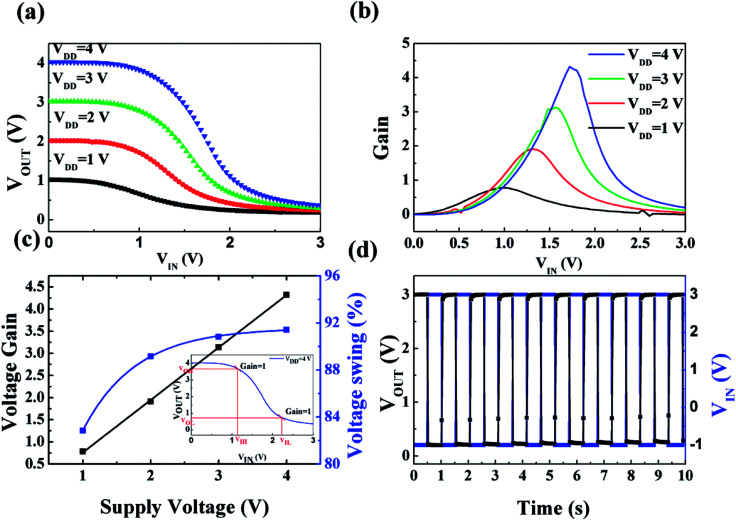
(a) The VTCs and (b) signal gain of resistor-loaded inverter coupled with 450 °C-IZO/HfAlO TFTs. (c) The voltage gain and the voltage swing of the inverter at various *V*_DD_ values. (d) Dynamic switching behavior of the inverter under square-waves at 1 Hz. The inset in (c) shows the noise margin calculation from VTC curve collected at 4 V *V*_DD_.

In spite of the improved electrical performance and traditional application in TFTs for solution-processed high-*k* gate dielectric films, the potential applications in more complex logic circuits were ignored. Encouraged by the excellent performance of 450 °C-derived IZO/HfAlO_*x*_ TFTs, their applications in inverter were further explored to demonstrate the logic operations.^53^ A simple unipolar resistor-loaded inverter was fabricated by coupling the 450 °C-annealed IZO/HfAlO_*x*_ TFTs with a 2.0 MΩ resistor. Fig. S6† shows the schematic diagrams and top views of the unipolar inverters, respectively. The *V*_in_, *V*_out_, and *V*_DD_ represent input voltage, output voltage, and supplied voltage, respectively. Fig. 11a shows voltage transfer characteristic (VTC) of the inverters logic circuit with the *V*_DD_ changing from 1 to 4.0 V. Distinct inverter characteristics are observed for the logic inverters at various *V*_DD_, in which the output voltage (*V*_out_) is switched between *V*_DD_ and 0 V when scanning input voltage (*V*_in_) from 0 to 3 V. If the *V*_in_ is 0 V, the inverter operates in the off-stage, resulting in *V*_out_ close to *V*_DD_. If the *V*_in_ is 3.0 V, the inverter turns on, resulting in *V*_out_ close to the ground level. Thus, when the *V*_in_ operates with a low and logical 0 signal, the *V*_out_ responds a high and logical 1 signal. Likewise, when the *V*_in_ operates with a high and logical 1 signal, the *V*_out_ responds a low and logical 0 signal. These results indicate that our logic circuit demonstrates full swing characteristics. The voltage gain, defined as −∂*V*_out_/∂*V*_in_, is displayed in Fig. 11b as a function of *V*_DD_. A linear dependence between the voltage gain and *V*_DD_ is observed. The maximum voltage gain of 4.46 has been obtained for *V*_DD_ at 4.0 V, which is larger than that of previous reported oxide TFT circuits at the same supply voltage.^55^ It should be noted here that a voltage gain of 2.5 is sufficient to drive the next stage component in a logic circuit.^55^ The voltage swing, which is defined as [*V*_out,max_ − *V*_out,min_]/*V*_DD_ × 100%, increases slightly from 82% to 91% when increasing *V*_DD_ from 1 to 4 V (Fig. 11c). The wider voltage swing of the inverter improves the noise margin characteristics, which makes the inverter run more reliably in the complex logic system. The improvement in voltage swing and gain with *V*_DD_ can be attributed to a high capacitive efficiency.^55^ Another important parameter is the noise margin, which is usually used to evaluate the multistage circuit operation reliability. The noise margin calculation from VTC collected at *V*_DD_ of 4 V is shown in the inset of Fig. 11c. The high (*N*_MH_) and low (*N*_ML_) noise margins of the inverter are defined as *N*_MH_ = *V*_OH_ − *V*_IH_ and *N*_ML_ = *V*_IL_ − *V*_OL_,^53^ where *V*_OH_ and *V*_OL_ represent output high and low voltage; *V*_IH_ and *V*_OL_ represent input high and low voltage, respectively, while ∂*V*_out_/∂*V*_in_ = −1. The *N*_MH_ and *N*_ML_ of this inverter are calculated to be 2.48 V and 1.55 V at *V*_DD_ of 4 V, revealing the potential application in multistage digital circuits.^56^ To investigate the alternative current (AC) characteristic of the inverter, the dynamic behavior at 1 Hz under AC square wave signal was measured, as shown in Fig. 11d. It can be noted that the as-constructed inverter exhibits good logic inversion action and responds well to the *V*_in_ square-wave signal, demonstrating its potential application in complex logic circuits, such as ring oscillators.

## 4 Conclusions

In summary, fully solution-processed IZO TFTs based on HfAlO_*x*_ dielectric have been fabricated successfully and annealing temperature dependent electrical properties of IZO/HfAlO_*x*_ TFTs has been investigated systematically. Amorphous HfAlO_*x*_ thin films annealed at 600 °C have shown a high transparency (>85%), low leakage current density (6.9 × 10^−9^ A cm^−2^ at 2 MV cm^−1^), and smooth surface. To verify the possible application of the HfAlO_*x*_ thin films as dielectrics in low-temperature-processed CMOS logics, fully solution-derived IZO/HfAlO_*x*_ TFTs have been successfully integrated to display a ultralow operating voltage of 3 V with optimized performance, including a high *μ*_sat_ of 5.17 cm^2^ V^−1^ S^−1^, an large *I*_on_/*I*_off_ of 7.5 × 10^6^, a small SS of 87 mV dec^−1^, an threshold voltage shift of 0.52 V under positive bias stress for 7200 s, respectively. To explore its potential application in complex logic circuits, a unipolar resistor-loaded inverter coupled with IZO/HfAlO_*x*_ TFT has been built and excellent swing characteristic and well dynamic behavior have been obtained. Our strategy opens a simple and reliable path toward fabricating low-cost, low-power consumption, and large-area environmentally friendly oxide flexible electronics.

## Conflicts of interest

The authors declare no competing financial interest.

## Supplementary Material

RA-008-C8RA07813K-s001
