# Supplementary material for: Potential solution-induced HfAlO dielectrics and their applications in low-voltage-operating transistors and high-gain inverters
Source: RSC Adv. 2018 Oct 30;8(64):36584–95. doi: 10.1039/c8ra07813k (PMC9088822; doi:10.1039/c8ra07813k)
Supplement: RA-008-C8RA07813K-s001 [file RA-008-C8RA07813K-s001.pdf]

Electronic Supplementary Material (ESI) for RSC Advances.
This journal is © The Royal Society of Chemistry 2018

# Potential solution-induced HfAlO dielectrics and their applications in low-voltage-operating transistors and high-gain inverters

Gang He[a,b*], Wendong Li[a], Zhaoqi Sun[a], Miao Zhang[a], and Xiaoshuang Chen[c]

[a]*School of physics and Materials Science, Radiation Detection Materials & Devices Lab, Anhui University, Hefei 230601, P.R. China*

[b]*Institute of Physical Science and Information Technology, Anhui University, Hefei 230601, P.R. China*

[c]*National Laboratory for Infrared Physics, Chinese Academy of Sciences, Shanghai Institute of Technical Physics, 500 Yutian Road, Shanghai 200083, P.R. China*

[*]Email: hegang@ahu.edu.cn

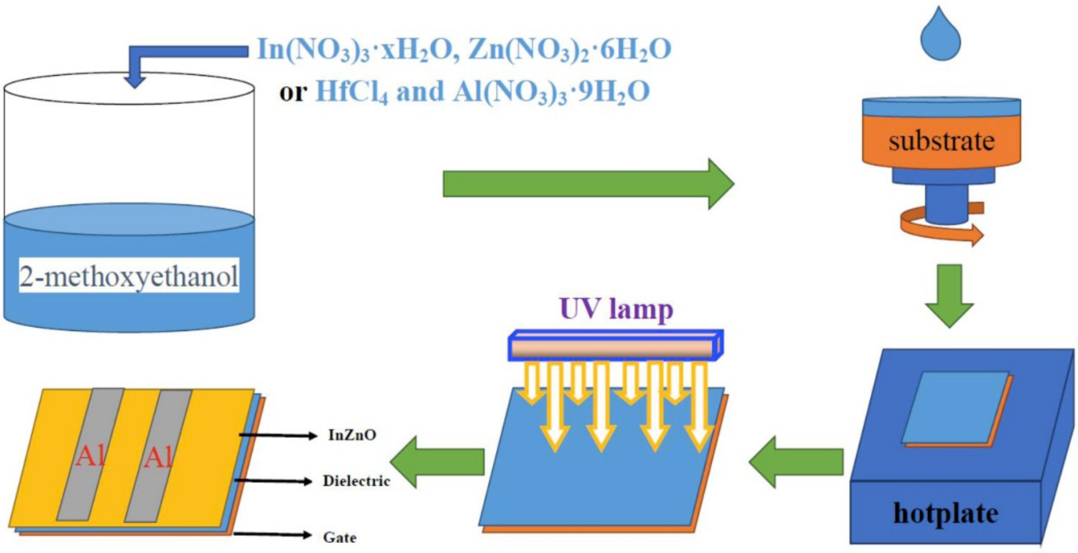


Fig. S1. Schematic diagram of solution-derived IZO and $HfAlO_x$ thin films and IZO TFTs device fabrication.

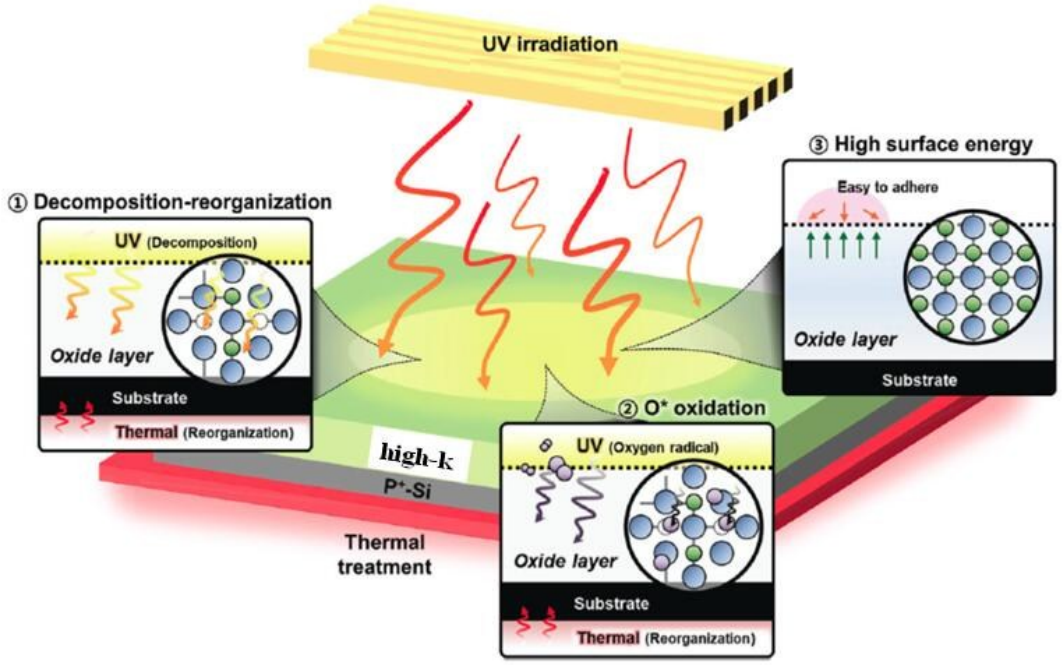


Fig. S2. Illustrated physical and chemical mechanisms of UV treatment.[1]

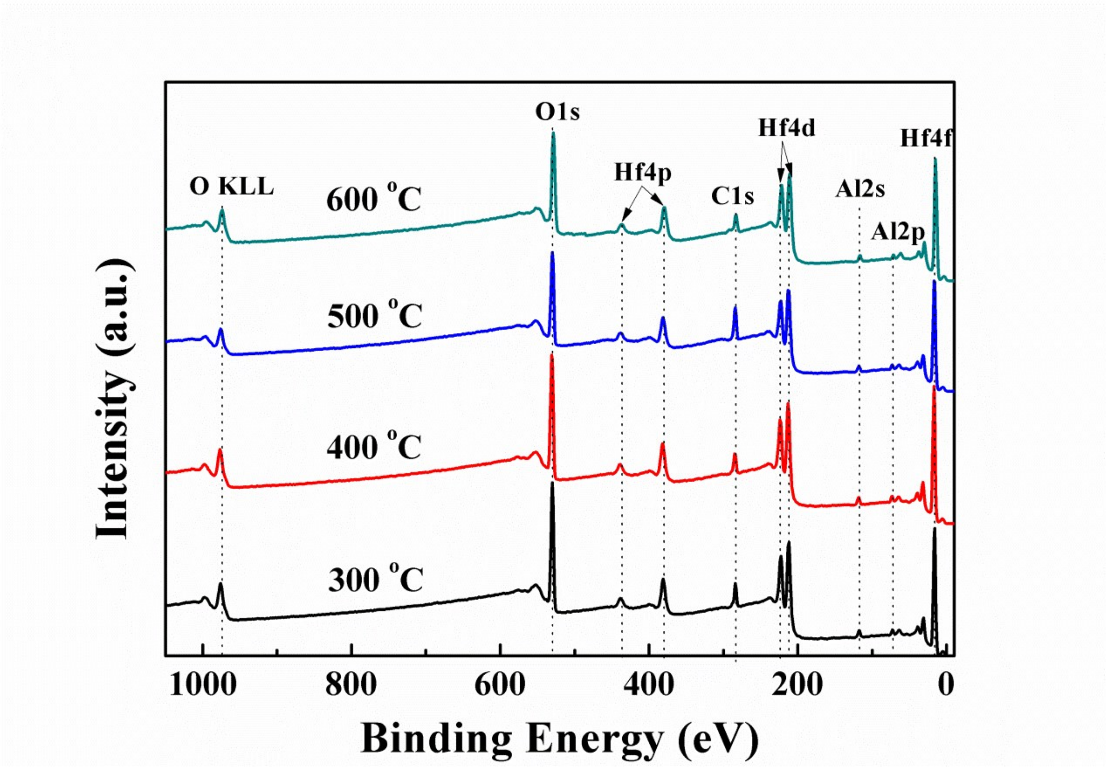


Fig. S3. XPS survey spectra of the $HfAlO_x$ films as a function of annealing temperature.

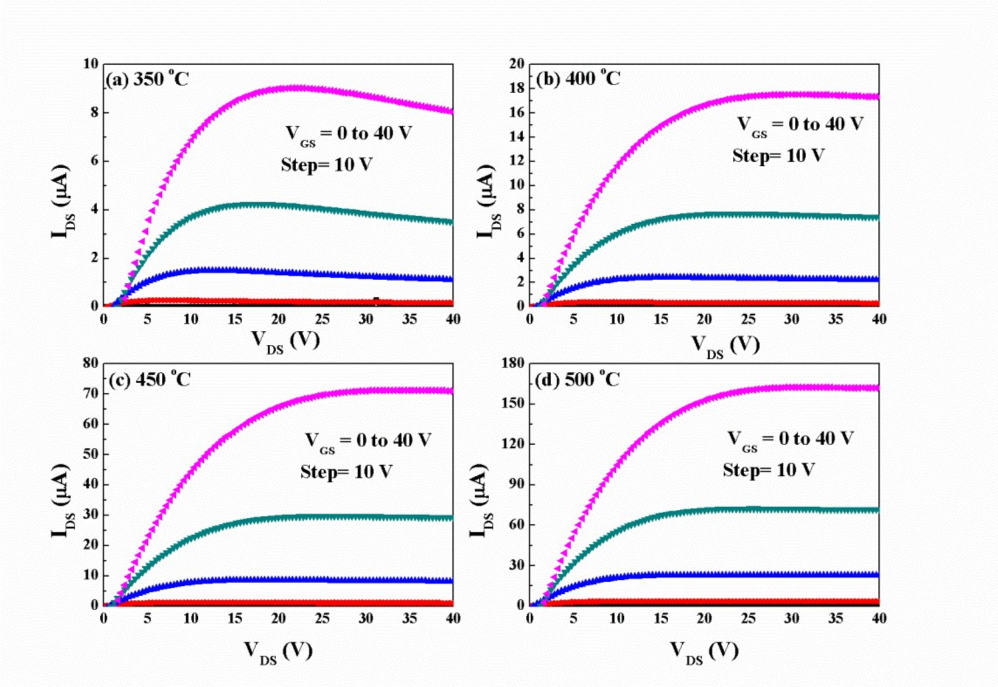


Fig. S4. The output characteristics of the IZO/$SiO_2$ TFTs as a function of annealing temperature.

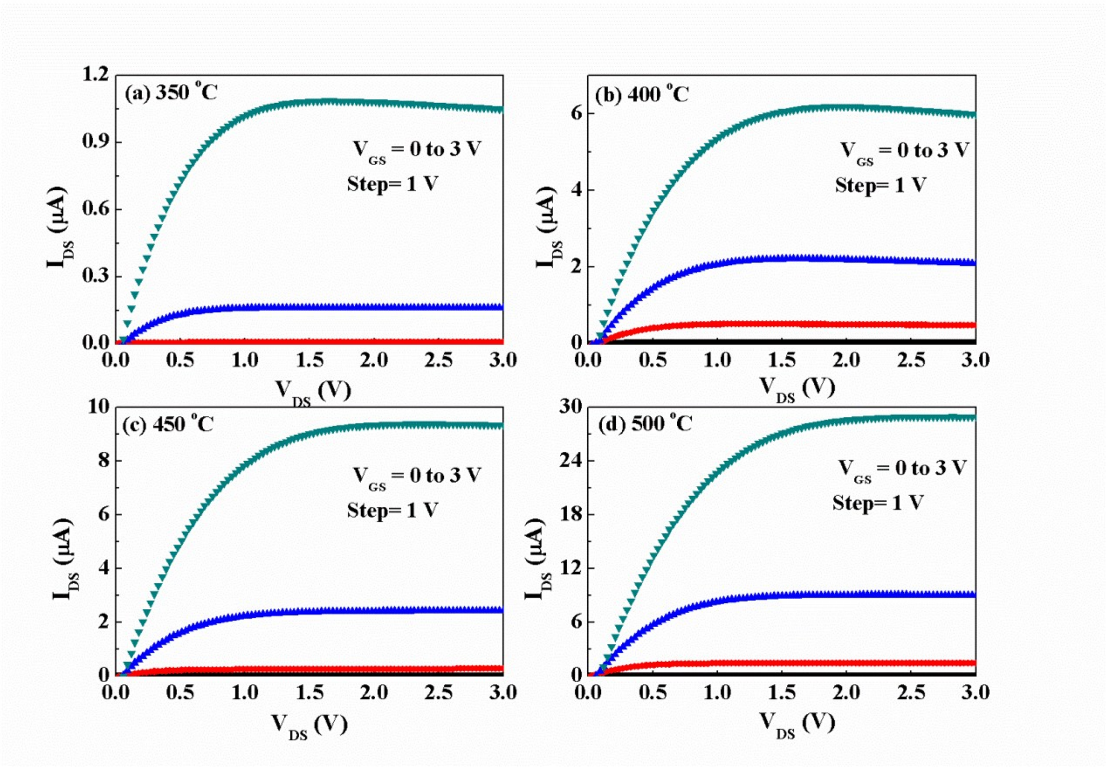


Fig. S5. The output characteristics of the IZO/$HfAlO_x$ TFTs as a function of annealing temperature.

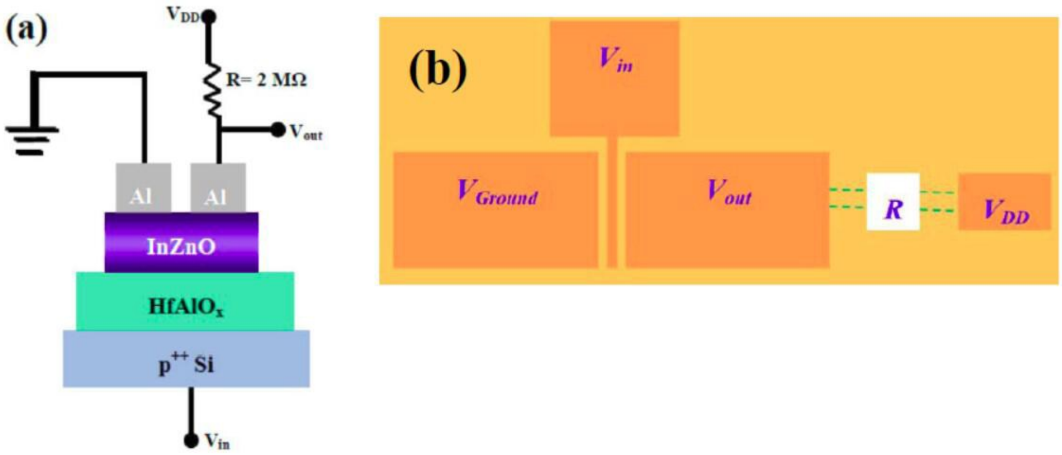


Fig. S6. The schematic diagrams (a) and top views (b) of the unipolar resistor-loaded inverter coupled with 450°C-annealed IZO/$HfAlO_x$ TFTs.

[1] Y. J. Tak, S. J. Kim. S. Kwon, H. J. Kim, K. B. Chung and H. J. Kim, *J. Mater. Chem. C*, **2018**, 6, 249.
